# Impact of an arbuscular mycorrhizal fungal inoculum and exogenous methyl jasmonate on the performance of tall fescue under saline-alkali condition

**DOI:** 10.3389/fmicb.2022.902667

**Published:** 2022-09-08

**Authors:** Hui Liu, Huimin Tang, Xiaozhen Ni, Yajie Zhang, Yingchao Wang

**Affiliations:** College of Life Sciences, Dezhou University, Dezhou, China

**Keywords:** arbuscular mycorrhizal fungi (AMF), methyl jasmonate (MeJA), saline-alkali stress, growth and physiology, tall fescue

## Abstract

Hormonal regulation and symbiotic relationships provide benefits for plants to overcome stress conditions. The aim of this study was to elucidate the effects of arbuscular mycorrhizal fungal (AMF) inoculum, methyl jasmonate (MeJA), and saline-alkali effects on the growth and physiology of tall fescue (*Festuca elata* “Crossfire II”). Treatments included AMF-inoculation, and non-AMF inoculation, four MeJA application concentrations (0, 50, 100, and 200 mg/L), and two saline-alkali levels (0 and 200 mmol/L). The results showed that AMF inoculation significantly enhanced saline-alkali resistance of the plants, and the beneficial effects were increased by MeJA at a concentration of 50 mg/L (50 MeJA) and decreased by MeJA at a concentration both of 100 (100 MeJA) and 200 mg/L (200 MeJA). AMF inoculation plants when treated with 50 MeJA accumulated significantly more biomass, had greater proline and total phenolic concentration, and lower malondialdehyde (MDA) concentration than plants only treated either with AMF or 50 MeJA. However, no significant differences in growth or physiological characteristics were observed between AMF and non-AMF plants when treated either with 100 or 200 MeJA. All of these results suggest that the interaction between a certain concentration of MeJA and AMF can significantly increase saline-alkali resistance of the tall fescue by regulating the biomass, proline, total phenolic, and MDA. Our findings provide new information on the effect of biological and chemical priming treatments on plant performance under saline-alkali stress.

## Introduction

Saline-alkali stress is one of the most important environmental factors limiting plant growth and productivity (Munns and Gilliham, 2015). Saline-alkali stress will have many adverse effects on plants, namely, osmotic stress, ion toxicity, and oxidative stress caused by salt stress (Chen et al., 2018; Szymańska et al., 2018). In addition, it causes further damage to plants due to the increase of pH under alkali stress. When the soil pH around the root system rises, some metal ions such as iron ions, magnesium ions, and calcium ions are deposited. With the decrease of inorganic anions, the absorption of mineral nutrients by plants is blocked, resulting in serious nutritional stress (Capula-Rodríguez et al., 2016; Guo et al., 2016). Therefore, greater insight is needed regarding mechanisms that mitigate those saline-alkali stress-induced ionic or oxidative damage.

Arbuscular mycorrhizal fungi (AMF) are a widely distributed microbial group in the terrestrial ecosystem, which can establish a symbiotic relationship with more than 80% of terrestrial vascular plant roots and form mycorrhizal structure (Gutjahr and Parniske, 2013). These symbiotic fungi help their host plants to reach water and nutrients, while they receive carbon products in turn, i.e., sugars and lipids. Arbuscular mycorrhizal fungi are considered essential elements for plant nutrition as their hyphae can extend for many meters in the ground, helping plants acquire mineral soil nutrients (Parniske, 2008; Balestrini and Lumini, 2018). Furthermore, the positive effects of AMF symbiosis on saline-alkali stress tolerance have been well-documented. For example, Wang et al. (2018) found that AMF enhanced the biomass and photosynthetic pigment contents, and improved the ion balance of *Leymus chinensis* under saline-alkali stress. Jia et al. (2019) suggested that mycorrhizal plants had a higher leaf soluble sugar and soluble protein content than that of the non-mycorrhizal plants during salt stress. Pan et al. (2020) in *Elaeagnus aangustifolia* showed that AMF inoculation significantly promoted the growth and improved soil nutrient uptake under saline soil. Qiu et al. (2020) demonstrated that AMF alleviated the detrimental effects of salt stress on *Ligustrum vicaryi* by mediating the N, Ca^2+^, Zn^2+^, Mg^2+^, and soluble protein content. Studies also found that AMF increased endogenous hormone concentration such as methyl jasmonate under abiotic stress (Zhang et al., 2018).

Methyl jasmonate (MeJA), is a naturally occurring plant growth regulator and participates in a variety of growth and development processes such as seed germination, root growth, fruit maturation, and aging (Mohamed and Latif, 2017). It can also activate plant defense mechanisms to deal with the damage caused by environmental stresses such as salinity, drought, low temperature, insects, and pathogens (Bastías et al., 2018; Ismail et al., 2018; Abdi et al., 2019; Khalvandi et al., 2019; Qin et al., 2019; Irankhah et al., 2020). Under salt stress, Ahmadi et al. (2018) showed that exogenously applied MeJA counteracted the inhibitory effects of salinity by increasing relative water content, soluble sugar content, and photosynthesis rate. Khalvandi et al. (2019) demonstrated that MeJA application ameliorated the negative effects of salinity on membrane electrolyte leakage and antioxidant activity. In addition, MeJA activated a variety of genes related to salt stress response, and improved plant salt tolerance by regulating reactive oxygen species and antioxidant enzyme systems (Ding et al., 2016). Although there are abundant studies on the interaction between plants and MeJA or AMF. However, there are few studies on the interactions between plant responses to MeJA and to AMF, especially under abiotic stress. Irankhah et al. (2020) found that growth parameters of *Trigonella foenum-graecum* were not strongly affected either by AMF or MeJA, but AMF in combination with MeJA significantly increased the diosgenin biosynthesis both under well-watered and water deficit growing conditions.

Tall fescue (*Festuca elata* “Crossfire II”) is a subtropical cool-season turfgrass that is an important forage resource widely used in lawn, stadium, and protective turf. It has better stress resistance and can be colonized by AMF (Li et al., 2021). Therefore, this study aims to determine the combined effect of AMF and MeJA on saline-alkali stress resistance of tall fescue. In particular, we addressed the following questions: (1) Does AMF inoculation have a positive effect on the resistance of tall fescue to saline-alkali stress? (2) Is there an interaction between AMF and MeJA treatment on the resistance of tall fescue to saline-alkali stress, and if so, is it antagonistic or synergistic?

## Materials and methods

### Experimental design

The experiment consisted of randomized complete block design with three factors: (1) inoculation (AMF, non-AMF), (2) methyl jasmonate (MeJA) treatment (0, 50, 100, and 200 mg/L), and (3) saline-alkali stress condition (0 and 200 mmol/L). Five replicates per treatment were used, requiring a total of 2 × 4 × 2 × 5 = 80 pots.

### Plant material and arbuscular mycorrhizal fungal inoculation

The tall fescue (*Festuca elata* “Crossfire II”) seeds were purchased from Clover Group Co., Ltd., Beijing, China. These were surface sterilized with 5% (v/v) hypochlorite and then washed with sterilized water for five times. Sterilized seeds were sown in pots, with 20 seeds per pot, filled with sterilized vermiculite. After germination, thinning to 15 uniform seedlings per pot (21 cm in diameter × 16 cm in height).

*Claroideoglomus etunicatum* was used in this study and prepared by trap culture in pots using Sorghum bicolor as the host plant under controlled greenhouse conditions for 3 months. Afterward, the soil was dried, and the roots were cut into <1 cm pieces, which were subsequently homogenously mixed together. Spore numbers were counted under a dissecting scope, and 100 g^–1^ inoculum containing ca. 300 AMF spores, demonstrating their viability. The amount of inoculum for *C. etunicatum* was 100 g per pot. The non-AMF treatment received 100 g of autoclaved inoculum and 50 ml of non-autoclaved inoculum filtrate (passed through a 10-μm sieve) to correct for possible differences in the microbial community between AMF and non-AMF treatments.

### Methyl jasmonate and saline-alkali stress treatment

Methyl jasmonate (purchased from Sigma) was dissolved in absolute ethanol and diluted in water to obtain a 50, 100, and 200 mg/L solution. Absolute ethanol was used as a control. Plants were sprayed until runoff of prepared MeJA 12 weeks after sowing. The purpose of setting different concentrations of MeJA is to find out the effective concentration that can alleviate saline-alkali stress. After exogenous MeJA treatment, saline-alkali treatment was carried out and contained two intensities: 0 and 200 mmol/L. Four salts NaCl, Na_2_SO_4_, NaHCO_3_, and Na_2_CO_3_ were mixed in a 9:1:1:9 M ratio to simulate mixed saline-alkali stress conditions according to the ion composition of saline-alkali soil in Northeast China. Plants were treated with modified 1/2 strength Hoagland nutrient solution (The nutrient solution contained: 2 mM KNO_3_, 0.5 mM NH_4_H_2_PO, 0.25 mM MgSO_4_^⋅^7H_2_O, 0.1 mM Ca(NO_3_)_2_^⋅^4H_2_O, 50 μM Fe-citrate^⋅^3H_2_O, 50 μM H_3_BO_3_, 10 μM MnCl_2_^⋅^4H_2_O, 1.6 μM ZnSO_4_^⋅^7H_2_O, 0.6 μM CuSO_4_^⋅^5H_2_O, and 0.05 μM (NH_4_) _6_Mo_7_O_24_^⋅^4H_2_O) supplemented with additional 0 and 200 mmol/L, and were dealt with 50 mmol/L saline-alkali on the first and second days to avoid saline-alkali shock. The saline-alkali levels determined in the experiment matched the range of natural environmental conditions without leading to extremely high mortality (Liu et al., 2022). The final concentrations of saline-alkali in each treatment were applied from day 3 onward. Every 3 days, the treatment solution was replaced to maintain consistent stress conditions. After 6 weeks of saline-alkali treatment, the shoots and roots were separated and harvested.

### Arbuscular mycorrhizal fungal colonization rate

A sub-sample (ca. 2 g) of roots was used to measure the AMF colonization, clearing in 10% KOH at 90°C in a water bath for 60 min, acidizing in 1% HCl for 3–5 min after cooling and staining in 1% trypan blue at 90°C in a water bath for 30 min (Phillips and Hayman, 1970). After destaining in glycerol–water, mycorrhizal colonization was determined by examining 40, 1 cm root segments using a compound microscope at ×200 magnification. The AMF colonization rate was recorded using the cross-hair eyepiece method (McGonigle et al., 1990), with a minimum of 200 intersections per pot.

### Growth and biomass

Shoots and roots were oven-dried at 80°C for 24 h and weighted for biomass determination. Ten fully expanded leaves growing on vegetative tillers per pot were chosen for measuring leaf area.

### Malondialdehyde, proline, and total phenolic concentrations

To determine the degree of oxidative stress, malondialdehyde (MDA), which is an indicator of membrane damage, was extracted and determined according to the method described by Puckette et al. (2007). Fresh leaf tissue (500 mg) was homogenized with 5 ml of 5% trichloroacetic acid and centrifuged at 3,000 rpm for 10 min. A 2 ml aliquot of the supernatant with 2 ml 0.67% (w/v) thiobarbituric acid was mixed, heated at 100°C for 30 min, cooled with ice, and then centrifuged at 3,000 rpm for 10 min. The absorbance of the supernatant was measured at 450, 532, and 600 nm. The calculation formula of MDA concentration was as follows: C = 6.45 (A_532_ – A_600_) - 0.56A_450_. Proline concentration was measured according to the colorimetric method described by Bates et al. (1973). In brief, 100 mg of dry leaf samples were homogenized in 10 ml 3% sulfosalicylic acid. A 2 ml homogenate was filtered and equal volume of 2 ml glacial acetic acid and 2 ml ninhydrin were added to the filtrate. The mixture was kept in a water bath at 100°C for 45 min. Then, proline was separated with 4 ml toluene, and the absorbance was read at 520 nm with toluene as blank. Total phenolic concentration of the shoot was determined by the method of Malinowski et al. (1998). About 200 mg samples of dry leaf material were weighed into 50 ml centrifuge tubes and extracted with 25 ml of ethanol and water (50:50). Tubes were shaken for 30 min and the extracts were filtered. About 1 ml of the supernatant and 49 ml of distilled water were added to a 150 ml flask and mixed thoroughly. To each flask containing the diluted supernatant and flasks with blank and standards, 6 ml of ferric chloride was added, and after 3 min, 6 ml of potassium ferricyanide was added. After a further 15 min, the absorbance of the sample and standards against blank were measured at 720 nm.

### Statistical analysis

Data were analyzed using SPSS 20.0 (SPSS Inc., Chicago, United States). First, two-way ANOVA was used to test the effects of saline-alkali stress and MeJA application on AMF colonization rate. Second, we tested the effects of saline-alkali stress, AMF inoculation, and MeJA application on the growth (biomass and leaf area) and physiological (MDA, proline and total phenolic) parameters using three-way ANOVA. When a significant effect was detected, the differences between means of different treatments were determined using Duncan’s multiple range tests at a probability of 0.05.

## Results

### Arbuscular mycorrhizal fungal colonization rate

**FIGURE 1:**
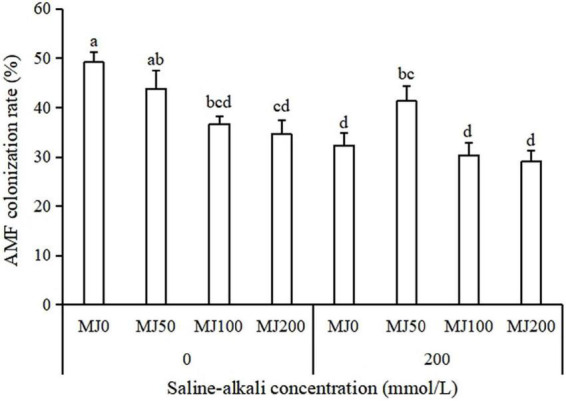
Arbuscular mycorrhizal fungi (AMF) colonization rate of tall fescue root as effects by the interaction between different methyl jasmonate (MeJA) levels (0 MeJA = 0 mg/L, 50 MeJA = 50 mg/L, 100 MeJA = 100 mg/L, 200 MeJA = 200 mg/L) and saline-alkali stress. Different letters above the bars indicate significant differences according to Duncan’s multiple range test (*P* ≤ 0.05).

The AMF colonization rate was significantly affected by saline-alkali stress, MeJA and the interaction of the saline-alkali stress and MeJA treatment (Table 1). Under non-saline-alkali conditions, MeJA application significantly decreased AMF colonization rate, and AMF colonization rate decreased with the increase of MeJA concentration. Under saline-alkali stress conditions, the AMF colonization rate appeared not to be strongly influenced by the MeJA with an exception of an increase of AMF colonization rate when treated with 50 MeJA (Figure 1).

**TABLE 1:** The ANOVA table for the AMF colonization rate, leaf area, shoot biomass, root biomass, MDA, proline, and total phenolic as impacted by arbuscular mycorrhizal fungi (AMF), methyl jasmonate (MeJA), saline-alkali stress (S), and their interaction of tall fescue.

	AMF colonization rate		Leaf area		Shoot biomass		Root biomass		MDA		Proline		Total phenolic	
	*F*	*P*	*F*	*P*	*F*	*P*	*F*	*P*	*F*	*P*	*F*	*P*	*F*	*P*
AMF			19.563	**<0.001**	23.800	**<0.001**	21.499	**<0.001**	21.374	**<0.001**	1.436	0.240	21.574	**<0.001**
MeJA	8.089	**<0.001**	52.628	**<0.001**	68.780	**<0.001**	27.780	**<0.001**	2.692	0.063	4.502	**0.010**	8.938	**<0.001**
S	17.778	**<0.001**	282.284	**<0.001**	548.168	**<0.001**	375.971	**<0.001**	82.697	**<0.001**	83.016	**<0.001**	150.947	**<0.001**
AMF × MeJA			2.773	**0.049**	2.806	**0.047**	2.763	**0.049**	3.363	**0.031**	2.409	0.085	0.521	0.671
AMF × S			8.279	**0.005**	7.233	**0.009**	7.636	**0.007**	10.314	**0.003**	1.879	0.180	10.994	**0.002**
MeJA × S	2.860	**0.043**	27.972	**<0.001**	20.396	**<0.001**	6.218	**0.001**	12.560	**<0.001**	4.632	**0.008**	4.857	**0.007**
AMF × MeJA × S			2.227	0.094	1.747	0.166	1.494	0.225	3.294	**0.033**	3.678	**0.022**	3.364	**0.031**

Significant P-values (P ≤ 0.05) are in bold.

### Growth parameters

**FIGURE 2:**
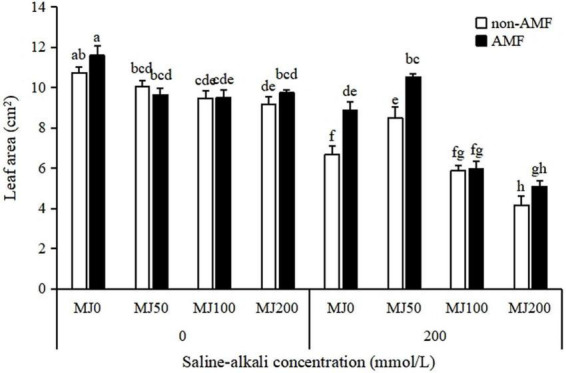
Leaf area of tall fescue as effects by the interaction between arbuscular mycorrhizal fungi (AMF), different methyl jasmonate (MeJA) levels (0 MeJA = 0 mg/L, 50 MeJA = 50 mg/L, 100 MeJA = 100 mg/L, 200 MeJA = 200 mg/L) and saline-alkali stress. Different letters above the bars indicate significant differences according to Duncan’s multiple range test (*P* ≤ 0.05).

Leaf area was strongly influenced by the “AMF × MeJA” interaction (Table 1). The leaf area under non-saline-alkali conditions was similar in AMF and non-AMF plants, regardless of MeJA application level. Under saline-alkali stress conditions, AMF inoculation was significantly enhanced by 33 and 24% leaf area when treated with 0 and 50 MeJA, respectively, but not either with 100 or 200 MeJA. A synergistic effect occurred between AMF and 50 MeJA, and the leaf area of AMF plants when treated with 50 MeJA was significantly greater than that of plants only treated with either AMF or 50 MeJA (Figure 2).

**FIGURE 3:**
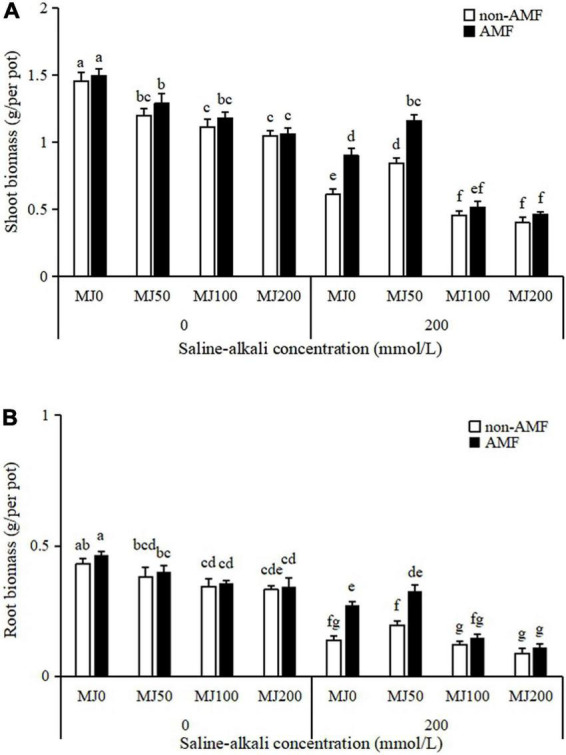
Shoot **(A)** and root **(B)** biomass of tall fescue as effects by the interaction between arbuscular mycorrhizal fungi (AMF), different methyl jasmonate (MeJA) levels (0 MeJA = 0 mg/L, 50 MeJA = 50 mg/L, 100 MeJA = 100 mg/L, 200 MeJA = 200 mg/L) and saline-alkali stress. Different letters above the bars indicate significant differences according to Duncan’s multiple range test (*P* ≤ 0.05).

Shoot and root biomass of plants were significantly affected by the AMF × MeJA interaction (Table 1). Under non-saline-alkali conditions, AMF inoculation had no significant effect either on the shoot or root biomass, regardless of MeJA application (Figure 3). Under saline-alkali stress conditions, shoot biomass was enhanced 38% when treated with 50 MeJA and decreased 46 and 53% when treated with 100 and 200 MeJA in non-AMF plants, respectively. Arbuscular mycorrhizal fungal inoculation enhanced shoot biomass as compared to non-AMF plants, and further enhancement was observed in these plants due to 50 MeJA application (Figure 3A). Unlike the shoot biomass, root biomass was increased 66% by AMF inoculation when treated with 50 MeJA, and this enhancement did not happen when treated either with 100 or 200 MeJA (Figure 3B).

### Malondialdehyde, proline, and total phenolic concentration

**FIGURE 4:**
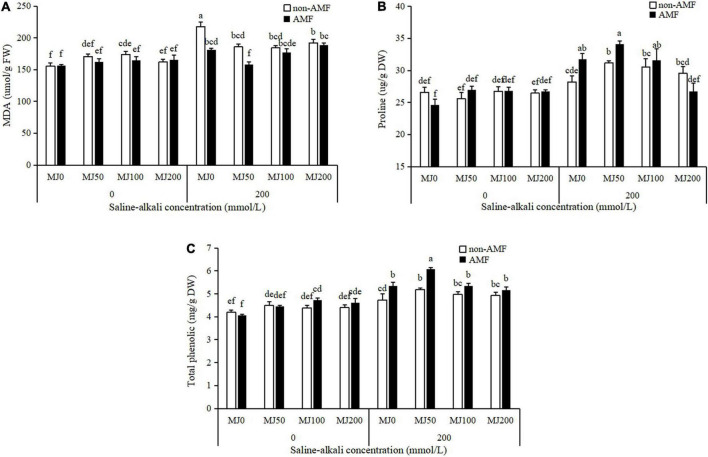
Malondialdehyde (MDA) **(A)**, proline **(B)**, and total phenolic **(C)** concentrations of tall fescue as effects by the interaction between arbuscular mycorrhizal fungi (AMF), different methyl jasmonate (MeJA) levels (0 MeJA = 0 mg/L, 50 MeJA = 50 mg/L, 100 MeJA = 100 mg/L, 200 MeJA = 200 mg/L) and saline-alkali stress. Different letters above the bars indicate significant differences according to Duncan’s multiple range test (*P* ≤ 0.05).

The interaction AMF × MeJA × saline-alkali stress has a significant impact on MDA concentration, as a marker of cellular damage (Table 1). Saline-alkali stress significantly increased MDA concentration. Both AMF inoculation and MeJA application mitigated the effect of saline-alkali on MDA, and a synergistic effect occurred between AMF and 50 MeJA. The concentration of MDA in plants simultaneously treated with AMF and 50 MeJA reached the lowest, compared to plants only treated either with AMF or 50 MeJA (Figure 4A).

Proline and total phenolic concentration were remarkedly influenced by the “AMF × MeJA × saline-alkali stress” interaction (Table 1). Under non-saline-alkali conditions, there were no obvious differences in proline and total phenolic concentration between AMF and non-AMF plants, regardless of MeJA application level (Figures 4B,C). Under saline-alkali stress conditions, AMF inoculation significantly increased 13 and 9% proline concentration, and 13 and 17% total phenolic concentration when treated with 0 and 50 MeJA, respectively, but not either with 100 or 200 MeJA (Figures 4B,C). In addition, a synergistic effect occurred between AMF and 50 MeJA on total phenolic concentration, and the concentration of total phenolic in plants simultaneously treated with AMF and 50 MeJA reached the highest, compared to plants only treated with either AMF or 50 MeJA (Figure 4C).

## Discussion

The results of this study showed, that saline-alkali stress has negative effects on the colonization of AMF. This reduction might be attributed to the inhibitory effect of saline-alkali stress on plant growth along with the reduction in the supply of carbon from the plant to AMF. Methyl jasmonate application hardly alleviated the detrimental effect of saline-alkali stress on the colonization rate of AMF. Only with one exception, AMF colonization rate was increased when treated with 50 MeJA. Sánchez-Romera et al. (2016) in *Phaseolus vulgaris* also demonstrated that there was no significant difference of AMF colonization rate between MeJA application and non-MeJA application treatments. Irankhah et al. (2020) showed that although the application of exogenous MeJA had no significant effects on AMF colonization rate, there was a positive correlation between MeJA application and arbuscular%, while some studies found that MeJA application has been reported to reduce the AMF colonization rate (Ludwig-Muller et al., 2002; Herrera-Medina et al., 2008).

Accumulating evidence suggests that AMF inoculation can promote plant growth and alleviate saline-alkali stress (Kong et al., 2019; Yang et al., 2020; Ci et al., 2021). Kong et al. (2019) found that AMF significantly promoted tomato growth, increased yield, and improved fruit quality under saline-alkali stress conditions. Yang et al. (2020) in *L. chinensis* showed that AMF inoculation significantly increased shoot, root, total dry weight, and shoot/root ratio under saline-alkali stress conditions. Ci et al. (2021) demonstrated that AMF in saline-alkali soil increased the main stem height, lateral branch length, and pod yield of peanuts to some extent. Consistent with previous findings, in our study, saline-alkali stress resulted in decreases in leaf area, shoot, and root biomass; however, AMF inoculation alleviated this growth inhibition. In addition, AMF inoculation plants had higher proline and total phenolic but lower MDA concentrations than non-AMF inoculation plants under saline-alkali stress conditions. Proline accumulation in plants exposed to saline-alkali stress may be a well-known adaptive mechanism for the maintenance of cell turgor pressure (Rossi et al., 2016), and MDA reduction implied that AMF inoculation plants were subjected to lower cell membrane damage than non-AMF inoculation plants under saline-alkali stress conditions. Total phenolic concentration was found a positive relationship with antioxidant activity (Khalvandi et al., 2019). In our study, AMF inoculation plants with higher total phenolic concentration, had higher antioxidant activity under saline-alkali stress conditions. The beneficial effects of AMF inoculation on saline-alkali stress tolerance in tall fescue through maintaining osmotic balance, stimulating antioxidant systems, reducing MDA concentration, and promoting growth.

It has been reported that the exogenous application of MeJA, either activates or inhibits plant growth under stress conditions depending on its applied concentration and plant species (Farooq et al., 2016). Similar results were observed in our study, we found that the adverse effect of saline-alkali stress on tall fescue was alleviated by 50 MeJA but not either by 100 or 200 MeJA. The ameliorating role of 50 MeJA associated with saline-alkali stress was mainly detected through increasing the growth (leaf area and biomass), proline, and total phenolic concentrations and by reducing the oxidative stress (MDA reduction). When MeJA was in combination with AMF, there was an AMF × MeJA interaction on tall fescue growth and physiology. The beneficial effect of AMF on plants was further enhanced by 50 MeJA under saline-alkali stress conditions, the biomass, proline, and total phenolic concentration of plants simultaneously treated by AMF and 50 MeJA reaching the highest, compared with the plants only treated with either AMF or 50 MeJA. Similar result was reported by Irankhah et al. (2020), which showed that secondary metabolite (diosgenin and trigonelline) accumulation reached the highest when MeJA was combined with AMF under the abiotic stress conditions. Sánchez-Romera et al. (2016) demonstrated that both AMF and MeJA counteracted the decrease in root hydraulic conductivity caused by abiotic stress treatment, but there was no interaction between AMF and MeJA.

## Conclusion

In general, there was an interaction between AMF and MeJA in enhancing the resistance of tall fescue to saline-alkali stress. A synergistic effect of AMF and 50 MeJA occurred on the growth and physiology of tall fescue under saline-alkali stress conditions. The effect of AMF combined with 50 MeJA on tall fescue biomass, proline, MDA, and total phenolic was better than that of AMF or 50 MeJA alone. However, there was no difference in tall fescue resistance to saline-alkali stress between non-AMF and AMF inoculation treatment when treated either with 100 or 200 MeJA. Our results provide some new insights that come out in symbioses- and MeJA-mediated saline-alkali stress responses in tall fescue that merit further investigations.

## Data availability statement

The raw data supporting the conclusions of this article will be made available by the authors, without undue reservation.

## Author contributions

HL designed the research and revised and polished the manuscript. HT, XN, YZ, and YW performed the experiments. HL and HT analyzed the data and wrote the manuscript. All authors contributed to the article and approved the submitted version.
